# Changes of Potent Odorants in Salted Duck Egg Yolk before and after Roasting

**DOI:** 10.3390/molecules29173984

**Published:** 2024-08-23

**Authors:** Xiaofan Hao, Miao Liang, Runhu Xin, Yuping Liu

**Affiliations:** School of Food and Health, Beijing Technology and Business University, Beijing 100048, China; 2103030104@st.btbu.edu.cn (X.H.); 2150031011@st.btbu.edu.cn (M.L.); 2230302043@st.btbu.edu.cn (R.X.)

**Keywords:** roasted duck egg yolk, odor-active compounds, aroma extract dilution assay, odor activity values, potent odorants

## Abstract

As the second most widely consumed eggs, duck eggs are made into preserved eggs, salted duck eggs, and roasted duck eggs to extend their shelf-life. To investigate the differences in potent odorants (POs) between salted duck egg yolk (SDEY) and roasted duck egg yolk (RDEY), the volatiles in SDEY and RDEY were extracted through solvent extraction coupled with solvent-assisted flavor evaporation and were assayed with gas chromatography-mass spectrometry-olfactometry. A total of 45 volatiles were identified in two samples, 24 odor-active compounds (OACs) were screened, and more OACs were in RDEY. The flavor-dilution (FD) factors of OACs were obtained by aroma extract dilution analysis and ranged from 3 to 6561. Twenty-two OACs with FD factors ≥ 9 were quantitated, and the results indicated the concentrations of OACs in yolk increased greatly after salted duck eggs were roasted. Based on the concentrations and thresholds, odor activity values (OAVs) were determined; 17 odorants with OAVs ≥ 1 were determined as POs. Acetoin was the most PO in SDEY; there were more POs in RDEY, including 2-ethyl-3,6-dimethylpyrazine, acetoin, 2-acetyl-3-methylthiophene, dihydro-4-hydroxy-2(3H)-furanone, etc. The outcomes obtained have reference values for making better use of duck eggs in the food industry.

## 1. Introduction

Being rich in proteins, lipids, phospholipids, fatty acids, and vitamins (A, B1, B2, D), etc. [1], poultry eggs are an important nutritious food in people’s daily lives. Among poultry eggs, duck eggs are the second most widely consumed eggs all over the world [2]. Because fresh duck eggs have an unpleasant fishy odor imparted by trimethylamine, they are rarely eaten directly by consumers [3]. Normally, most fresh duck eggs are treated to remove the off-odor and form the pleasant odor and then sold in the market. There are three methods for treating fresh duck eggs. The first is that eggs are processed into preserved eggs called pidan [4], which have a special and unusual odor and are liked by some people; the second is that the eggs are brined into salted duck eggs (SDE) [5]; the last one is that eggs are brined and then roasted to obtain roasted duck eggs (RDE) [6]. Generally, fat combines with protein in duck egg yolk. After being brined and roasted, fat separates from protein, and riboflavin and carotene dissolve into fat, which makes duck egg yolk take on an orange color [7]. RDE white has an elastic texture like meat, and RDE yolk has an attractive color and flavor, so it is favored by many people. Moreover, this processing method could prolong the shelf-life of duck eggs.

Food aroma is one of the important attributes of food and has a great influence on the acceptance of food for many consumers. Of the three kinds of processed duck eggs, more people prefer RDE, but there is not a report on the odor of RDE. At present, the investigations on duck egg odor mainly focused on the analyses of volatile compounds in fresh raw duck eggs [8], boiled duck eggs [9], duck egg yolk [10], salty duck egg white and egg yolk [5,11], preserved egg white and yolk [12,13,14], and fishy odor in duck egg yolks [3]. In most of these reports published, simultaneous distillation extraction (SDE) [9,12,13] and solid phase microextraction (SPME) [5,8,10,11] were mainly used for isolating the volatiles from duck eggs; solvent-assisted flavor evaporation (SAFE), regarded as one of the best isolation methods, was seldom used in isolating the volatiles of duck eggs [14]. Additionally, the volatiles were only identified tentatively by mass spectrum (MS) and retention index (RI) or only by MS in these references, and the qualitative analysis results needed to be confirmed further. Moreover, the volatiles were quantitated simply by peak area normalization, and their concentrations were shown by relative content.

Not all volatile compounds contribute to the overall odor profile of duck eggs; the compounds contributing to the odor are key odorants. Up to now, the key odorants in preserved egg yolk had been characterized by SPME and SAFE coupled with gas chromatography-olfactometry (GC-O) and gas chromatography-mass spectrometry (GC-MS) analyses as well as the calculations of odor activity values (OAV, the ratio of the concentration of an aroma compound to its odor threshold), and these compounds primarily belonged to aldehydes and sulfur-containing compounds [14]. In addition, 1-octen-3-ol, (*E*)-2-octenal, hexanal, (*E*, *E*)-2,4-decadienal and 2-pentylfuran were identified as key off-odor components in thermal duck egg gels; the results also showed that the off-odor of thermal duck egg was mainly from the egg yolk, and anaerobic treatment in nitrogen could reduce off-odor [15]. However, the key odorants in roasted duck egg yolk have not been characterized at present.

Since the odor compounds are mostly found in the yolk of duck eggs, the objectives of the present research are to (i) identify volatiles and odor-active compounds in salted duck egg yolk (SDEY) and roasted duck egg yolk (RDEY) by gas chromatography-mass spectrometry-olfactometry (GC-MS-O), (ii) to screen the important odor-active compounds in SDEY and RDEY by aroma extract dilution analysis (AEDA), (iii) to quantitate the important odor-active compounds, (iv) to determine the potent odorants by calculating OAVs, and (v) to measure the fatty acid and amino acid concentrations in SDEY and RDEY.

## 2. Results and Discussions

### 2.1. Identification of Volatile Compounds in SDEY and RDEY

The overall odor profiles of both SDEY isolate and RDEY isolate obtained by SE-SAFE were assessed by placing a drop of the condensed isolate on an odorless strip of blotting paper and sniffing the isolate at certain intervals by three flavorists; the results indicated that two isolates had the same odor characteristics as SDEY and RDEY, respectively, which meant odor compounds were isolated successfully from the yolk by SE-SAFE. Additionally, the odor intensity of the RDEY isolate was stronger than that of the SDEY isolate. Subsequently, two isolates were assayed by GC-MS-O on both DB-wax column and HP-5 column, and more volatile substants were separated on DB-wax column. A total of 45 compounds (Table 1) were characterized, including seven pyrazines, seven compounds containing thiophene rings, seven compounds containing furan rings, five alcohols, four aldehydes, four ketones, three acids, two phenols, one ester, and five other compounds. Eighteen volatile compounds were identified in SDEY, and 43 volatiles in RDEY; 16 volatiles were common in both SDEY and RDEY. That is to say, nearly all of the volatiles identified in SDEY could be found in RDEY. It was worth noting that only seven pyrazines and seven compounds containing thiophene rings were identified in RDEY, which showed that more volatiles could be formed after SDEs were roasted. Of 45 volatiles identified, only benzaldehyde and 2-pentylfuran had been identified in fresh duck egg yolk and SDEY by SPME coupled with GC-MS [10]; 1-octen-3-ol, benzaldehyde, 2-pentylfuran, nonanal and dibutyl phthalate were found in SDEY lipid by SPME combined with GC-MS [11]; pyrazine, methylpyrazine, 2,6-dimethylpyrazine, hexanal, benzaldehyde, nonanal, 1-octen-3-ol, indole, 2-acetythiazole, and 2-pentylfuran were identified in preserved duck egg yolk by SE-SAFE coupled with GC-MS and GC-O [14]. Except for the volatiles mentioned above, the other compounds had not been reported in fresh duck egg yolk and treated duck egg yolk in the published references.

### 2.2. Odor-Active Compounds Screened from the Volatiles in SDEY and RDEY

To screen the odor-active substances from the volatiles identified in SDEY and RDEY, the two isolates were analyzed further by GC-MS-O. The results were listed in Table 2. It could be found that only 24 compounds out of 45 volatiles were odor-active, including five pyrazines (2, 5, 6, 7, 9), five containing furan cycle compounds (16, 17, 19, 23, 24), five alcohols (8, 11, 12, 14), three containing thiophene ring compounds (1, 13, 18), two hydroxyl ketones (3, 4), and five other compounds (10, 15, 20, 21, 22). Ten odorants were identified in SDEY and 23 compounds in RDEY; nine odorants were common in two samples. To further determine more important odor-active compounds, the flavor-dilution (FD) factors were determined by AEDA, and the results were also listed in Table 2. On the whole, the FD factors of these odorants ranged from 3 to 6561; their values in RDEY were higher than those in SDEY.

In SDEY, 1-octen-3-ol (mushroom, earth) and dihydro-5-hydroxymethyl-2(3*H*)-furanone (burnt) manifested the highest FD factor of 729, followed by 2,4-di-tert-butylphenol (phenolic odor) with an FD factor of 243 as well as by acetoin (butter, creamy), *β*-butoxyethanol (sweet, burnt), and 2-ethyl-1-hexanol (green, rose) with an FD factor of 81. In RDEY, 1-hydroxy-2-propanone (butter, malty) and 3-hydroxy-4,4-dimethyldihydro-2(3*H*)-furanone (sweet, caramel) yielded the highest FD factor of 6561, followed by dihydro-5-hydroxymethyl-2(3*H*)-furanone (burnt) with an FD factor of 2187, 2,4-di-tert-butylphenol and (*Z*)-9-octadecenal (fatty) with an FD factor of 729, as well as by 2-ethyl-3,6-dimethylpyrazine (earth, potato, roasted), 2-ethyl-1-hexanol, carbitol (sweet, burnt), 2-furanmethanol (burnt, caramel, cooked), 2-acetyl-3-methylthiophene (creamy), indole (fecal, jasmine), and dihydro-4-hydroxy-2-(3*H*)-furanone (caramel) with an FD factor of 243.

After roasting, more categories of odor-active constituents were produced. From the structures of these newly formed odorants, it could be known that the formations of most of them were associated with Maillard reaction (MR). To prove the conjecture, the concentrations of free amino acids in SDEY and RDEY were measured (Table 3), and the results indicated that total levels of free amino acids in yolk increased after the duck eggs were roasted. Duck egg yolk contained not only free amino acids but also carbohydrates [16,17], and the temperature of roasting duck egg was above 100 °C [6], which was beneficial for MR. Pyrazines could be formed by α-amino acids, α-dicarbonyl compounds, and carbohydrates; pyrazine and 2,5-dimethylpyrazine were found in baked goods [18]. In the mixture of MR product of glucose and glutamine-amide, eight pyrazines, including pyrazine, 2,5-dimethylpyrazine, 2,6-dimethylpyrazine, 2-ethyl-5-methylpyrazine, etc., were identified, and the yields of pyrazines in the dry heating system were higher than those in the aqueous system [19]. In the volatiles of the MR mixture of L-ascorbic acid and L-alanine, many pyrazine compounds including 2,5-dimethylpyrazine, 2-ethyl-5-methylpyrazine, etc., were also detected [20]. The formations of thiophene compounds were related to MR, including sulfur-containing amino acids; for example, 2-methylthiophene was identified in MR fragrance compounds of a cysteine–xylose model system [21], and dihydro-2-methyl-3(2*H*)-thiophenone was found in MR mixture of cysteine and thiamine with xylose [22]. 2-Acetylthiazole was detected in the model reaction of D-glucose and L-cysteine, and its formation route was regarded as the reaction of glyoxal and methylglyoxal produced by D-glucose with hydrogen sulfide and ammonia derived from L-cysteine [23]. In L-alanine/glucose Maillard model systems, acetoin and 1-hydroxy-2-propanone were detected, and it was proved that most of 1-hydroxy-2-propanone (70%) was from a retro aldol cleavage of isomerized 1-deoxyglucosone [24]. 2-Furanmethanol was generated from 2-furfural, which could be formed in MR between glucose and cysteine or glucose alone [25]; 5-methyl-2-furanmethanol was also identified in amino compound-glucose MR models [26]. It was noteworthy that very few odor-active compounds from the degradation of fatty acid were found in RDEY; only (*Z*)-9-octadecenal was detected. The reason might be that duck egg yolk was surrounded by egg white, which was covered by eggshell; this structure could prevent oxygen from entering egg yolk and reduce the oxidation of fat in duck egg yolk during roasting. To attest the guesswork, the concentrations of fatty acids in SDEY oil and RDEY oil were analyzed (Table 4), and the results indicated total contents of fatty acids in SDEY oil and RDEY oil changed slightly.

### 2.3. Quantitation of Important Odor-Active Constituents in SDEY and RDEY

To calculate the OAVs, a total of 22 odor-active compounds with FD factors ≥ 9 in SDEY and RDEY were quantitated (Table 5). The totals of concentrations of odor-active compounds in RDEY were higher than those in SDEY, which meant that roasting could increase the contents of odorants. Among seven common constituents in the two samples, the content of acetoin in RDEY was lower than that in SDEY. As a precursor, acetoin could be converted into new compounds, such as diacetyl, 2,3-butanediol, pyrazines, esters, etc. [27] Except for acetoin, the other common odorants had more concentrations in RDEY; the contents in RDEY were over tenfold higher than those in SDEY.

In SDEY, 10 odorants were quantitated; acetoin had the highest content, followed by dihydro-5-hydroxymethyl-2(3*H*)-furanone, 2,4-di-tert-butylphenol, acetic acid, dihydro-4-hydroxy-2(3*H*)-furanone, 2-ethyl-1-hexanol, and *β*-butoxyethanol; the contents of the other odorants were less than 5 mg/kg.

In RDEY, 19 odorants were quantitated, and dihydro-4-hydroxy-2(3*H*)-furanone had the highest concentration, followed by 1-hydroxy-2-propanone, dihydro-5-hydroxymethyl-2(3*H*)-furanone, pyrazine, 2-acetyl-3-methylthiophene, 2,4-di-tert-butylphenol, and 2-ethyl-3,6-dimethylpyrazine. Although the levels of the other 12 odor substances were less than 100 mg/kg, their contents were higher than 1 mg/kg.

### 2.4. Calculations of OAVs

To further assess the contributions of the quantitated odor-active compound to the characteristic odor of SDEY and RDEY, OAVs of the odorants were measured based on the concentrations in Table 5 and their thresholds in water [28], and the results obtained were shown in Table 6. Of the nine odorants in SDEY, six odor-active compounds yielded OAVs ≥ 1, and they should contribute to the characteristic odor SDEY. Acetoin showed the highest OAV = 9737, followed by 1-octen-3-ol and 2,4-di-tert-butylphenol. Although the other four odors had OAVs ≥ 1, all of the values were less than 35; they had fewer contributions to the odor of SDEY. From the OAV results, it could be known that the characteristic odor of SDEY was imparted mainly by acetoin and 1-octen-3-ol, which contributed to butter, creamy, raw mushroom, and earthy notes.

Of the 15 odorants in RDEY, all of them yielded OAVs ≥ 1, showing they were the potent odorants. 2-Ethyl-3,6-dimethylpyrazine had the highest OAV = 14,766, followed by acetoin and 2-acetyl-3-methylthiophene; they were the most important fragrance compounds contributing to the overall odor profile of RDEY. The odorants with OAVs = 107–698 included 1-hydroxy-2-propanone, pyrazine, 2,4-di-tert-butylphenol, and dihydro-4-hydroxy-2(3*H*)-furanone, and were moderate potency odorants. 2-Ethyl-1-hexanol, carbitol, 2-furanmethanol, 2,6-dimethylpyrazine, 2,5-dimethylpyrazine, and 2-ethyl-5-methylpyrazine, as well as indole, had the OAV = 12–88; they also had a few contributions to the characteristic odor profile of RDEY. 2-Methylthiophene had an OAV = 2, and it contributed less to the odor of RDEY. Combing the odor of these odorants with their OAV, it could be deduced that the characteristic odor of RDEY was mainly related to ethyl-3,6-dimethylpyrazine, acetoin, 2-acetyl-3-methylthiophene, and dihydro-4-hydroxy-2(3*H*)-furanone; they imparted RDEY roasty, butter, creamy, caramel notes. The results can be used for improving the processing conditions of roasted duck eggs, and they also provide a theoretical basis for making better use of duck eggs in the food industry.

## 3. Materials and Methods

### 3.1. Materials

The experimental samples including SDEs and RDEs were obtained from Jiangsu Fuyou Food Co., Ltd. (Nanjing, China). All samples were produced in the same batch, and they were stored in a refrigerator under 4 °C prior to experiments.

### 3.2. Chemicals

2-Thiophenemethanol (97%), 2-methylthiophene (99%), 3-acetylthiophene (98%), 5-methyl-2-furanmethanol (97%), 2-hydroxy-2-methylpropanoic acid (98%), 2-acetyl-3-methylthiophene (97%), 2-cyclopenten-1-one (97%), dibutyl phthalate (98%), dihydro-2-methyl-3(2*H*)-thiophenone (97%), *β*-butoxyethanol (99%), carbitol (98%), thiophene (99%), 2-pentylfuran (98%), pyrrole (99%), hexanal (97%), phenylethyl alcohol (98%), indole (99.5%), meta-methoxybenzenethiol (> 97%), 7,9-di-tert-butyl-1-oxaspiro(4,5)deca-6,9-diene-2,8-dione (97%), (*Z*)-9-octadecenal (95%), and pyrogallic were received from Macklin Co., Ltd. (Shanghai, China). 2,5-Dimethylpyrazine (98%), acetic acid (99%), 2,6-dimethylpyrazine (98%), 2-furanmethanol (98%), 2-hydroxy-3-methyl-2-cyclopenten-1-one (99%), 2,4-di-tert-butylphenol (99%), dihydro-5-hydroxymethyl-2(3*H*)-furanone (95%), methylpyrazine (98%), ethylpyrazine (98%), 2-acetylthiazole (99%), dimethyl sulfone (99%), dihydro-3-(2*H*)-thiophenone (98%), and n-hexadecanoic acid(97.5%) were received from J & K Chemical Ltd. (Beijing, China). 1-Hydroxy-2-propanone (80%), phenol (99.5%), nonanal (95%), 3-hydroxy-4,4-dimethyldihydro-2(3*H*)-furanone (95%), and 2-ethyl-1-hexanol (99%) were bought from TCI (Shanghai, China). 2-Ethyl-5-methylpyrazine (97%), acetoin (97%), 1-octen-3-ol (>97%) were purchased from Adamas Reagent Co., Ltd. (Shanghai, China). Dihydro-4-hydroxy-2-(3*H*)-furanone (96%) and 2-ethyl-3,6-dimethylpyrazine (99%) were obtained from Alfa Aesar Chemical Co., Ltd. (Shanghai, China). Benzaldehyde (95%) was gained from Beijing Zoteq Co., Ltd. (Beijing, China). Pyrazine (99%) and sodium sulfate anhydrous (99%) were obtained from Aladdin Reagents Co., Ltd. (Shanghai, China). Glyceryl triundecanoate (98%), ethanol (99%), *n*-pentane (95%), *n*-heptane (99%) and *n*-hexane (95%) were obtained from Innochem Co., Ltd. (Shanghai, China). *n*-Alkanes (C7–C28) were provided from Aldrich Chemical Co., Ltd. (Shanghai, China).

### 3.3. Sample Pretreatment

For RDEs, RDEY was separated from egg white, put into an odorless glass bottle, and then stirred by a glass rod to obtain the homogenic slurry for the following experiments. For raw SDEs, firstly, they were taken out from the refrigerator and heated in an egg steamer for 10 min to make egg white coagulate, and then SDEY was isolated and treated as above.

### 3.4. Isolation of Volatiles of RDEY (or SDEY) by Solvent Extraction Coupled with Solvent-Assisted Flavor Evaporation (SE-SAFE)

SE-SAFE was applied to isolate the volatiles in RDEY based on the reported method with some modifications [14]. Firstly, 35 g RDEY (or SDEY) was merged with 80 mL dichloromethane in a 250-mL flask, and followed by shaking at room temperature in an oscillator (ZWY-100H, Shanghai ZAIM Co., Ltd., Shanghai, China) at 180 rpm for 1 h. Afterwards, the mixture was treated to obtain filtrate and filter cake; the latter, filter cake, was washed twice with 80 mL dichloromethane as above. Three filtrates were combined and subjected to SAFE under a pressure of 2.5 × 10^−5^ mbar (Edwards TIC Pumping Station from BOC Edwards, Crawley, UK) to isolate the volatiles. The distillate obtained was dehydrated with anhydrous NaSO4, then condensed to ~5 mL using a Vigreux glass column (50 cm), and finally condensed to ~100 μL by use of a nitrogen stream. The condensed distillate was kept at a temperature of −20 °C until GC-MS-O analyses were performed.

### 3.5. GC-MS-O Analysis

GC-MS-O analyses were performed on a 7890B GC coupled with a 5975 mass detector (Agilent Technologies Inc., Santa Clara, CA, USA) and an olfactometer (ODP3 Gerstel, Mülheim an der Ruhr, Germany). The condensed distillate obtained above was analyzed not only on a DB-Wax column but also on an HP-5MS column (30 m × 0.25 mm i.d × 0.25 μm film, Agilent Technologies) under the carrier gas, which was helium with a flow speed of 1.7 mL/min. The temperature of GC injection inlet was maintained at 250 °C; the spitless mode was set, and the injected volume was 3 µL. The temperature of the column box was held at 35 °C (maintained for 2 min), rose to 45 °C at a speed of 3 °C/min, and then ramped to 120 °C at a speed of 2 °C/min; it further increased to 230 °C at a speed of 6 °C/min and was held for 5 min. The effluent from the chromatographic column was separated into two parts with equal volume by a Y-type splitter. One part entered MS, the other went to O.

Under ionization energy of 70 eV, mass spectra were obtained. The temperatures of the quadrupole and ion source were kept at 150 °C and 230 °C, respectively. The ions of *m*/*z* 33–350 were collected under full-scan mode.

The transfer line temperature from GC to O was 250 °C, and the temperature of the olfactory port was maintained at 220 °C. To keep the sensitivity of the evaluator’s nose during GC-MS-O analyses, the smelling port was coupled with moist air. GC-MS-O analysis was carried out by three students majoring in flavor and fragrance at Beijing Technology & Business University, who had been trained to smell the odor nature of odorants for more than 1 year. A fragrance location was determined when the fragrance was sniffed by at least two evaluators.

### 3.6. Qualitative Analysis of Odor-Active Compounds

The odorants were characterized by matching their odor qualities, MS data, and RI with the relevant data of the corresponding standard substances. If standard substances couldn’t be obtained, they were merely identified tentatively by matching their odor descriptions, MS data, and RI with the corresponding information in the databases and reported in the published literatures. RI was calculated according to the retention times of measured normal alkanes, referring to the report from Van Den Dool, & Kratz [29].

### 3.7. Aroma Extract Dilution Analysis (AEDA)

The condensed isolate obtained from Section 2.4 was triple diluted with redistilled dichloromethane (1:3, 1:9, 1:27, …, 1:6561), and then each of diluted isolates was assayed by GC-MS-O until the odor of odorants was no longer detectable. At least two AEDA tests were conducted for each sample, and the maximum dilution of original isolate in which odorants could be smelled by evaluators was regarded as the flavor-dilution (FD) factor.

### 3.8. Quantitative Analysis of Odor-Active Compounds

The quantitative analysis experiment was performed by means of the internal standard (IS) method. Based on the criteria that the internal standard was not present in the sample and did not react chemically with the analytes and could be separated from each component in the samples, three ISs including 2-isopropylphenol, 2-octanol, and 2-methyl-3-heptanone were selected. The contents of the internal standard added were based on preliminary experiments and were close to those of the analytes. Under the same GC-MS conditions utilized in Section 3.5, the selected ion-monitoring mode was employed, and quantitative analyses were conducted repeatedly three times. The concentrations of odorants quantitated were calculated by the following formula:

$$c=\frac{fSC_{IS}}{S_{IS}}$$

where *c* means the content of a fragrance compound; *f* means the relative correction factor of an odorant to its IS, which is measured by analyzing the odor-active compound and IS with equal concentration; *S* means the peak area of the odorant; *C_IS_* means the concentration of IS in the sample; and *S_IS_* means the peak area of IS.

### 3.9. Calculation of OAV

The OAV of each odor-active composition was the ratio of its content in RDEY or SDEY to its threshold value in water. It was determined that the odorant with OAV ≥ 1 made a great contribution to the sample’s overall odor profile. 

### 3.10. Analysis of Free Amino Acids

The analysis of free amino acids in RDEY or SDEY filter cakes obtained in Section 2.4 was carried out according to the method in the Agela Technology Durashell AA Amino Acid Analysis Manual by an Agilent 1260 series high-performance liquid chromatography (HPLC), which included a degasser, quaternary pump, auto-injector, and DAD detector [30]. The chromatographic column was a Durashell AA column, whose particle size, inner diameter, and length were 3 µm, 4.6 mm, and 150 mm, respectively. The column temperature was kept at 45 °C. Mobile phase (MP) A consisted of a solution of Na_2_HPO_4_·12H_2_O (9.0 g), Na_2_B_4_O_7_·10H_2_O (9.5 g), and water (to 2000 mL), and then pH value was regulated to 8.2 using 36% hydrochloric acid (approximately 3 mL). A 0.45 µm membrane filter was used to filter the solution prepared above. MP B was obtained by mixing methanol (450 mL), acetonitrile (450 mL), and water (100 mL). The mixture was sonicated to remove bubbles. The elution conditions and flow rates were as follows: 0–6 min, MP B raised from 6% to 10% at a flow velocity of 1.6 mL/min with a 2 min holding; 8–10 min, MP B ramped from 10% to 16% at a lower flow velocity of 1.3 mL/min; 10–23 min, MP B ramped from 16% to 40% at a reduced flow speed of 1.0 mL/min; 23–30 min, MP B raised from 40% to 50% at a higher flow speed of 1.6 mL/min; 30–31 min, MP B increased from 50% to 100% with the flow velocity of 1.6 mL/min; held for 3 min; and finally, MP B reduced from 100% to 6% at a fixed flow velocity of 1.6 mL/min and held for another 3 min.

### 3.11. Determination of Fatty Acids

RDEY (or SDEY) were extracted by dichloromethane and n-hexane, respectively. The merged extraction solution was subjected to SAFE, and egg yolk oil was obtained. According to GB 5009.168-2016 China National Food Safety Standards Determination of Fatty Acids in Food [31], fatty acids in RDEY and SDEY were analyzed. Egg yolk oil (0.2 g) was mixed with 1 mL IS solution (heptadecanoic acid triglyceride, 5 mg/mL), and the mixture was treated to obtain fatty acid methyl esters (FAMEs). To analyze FAMEs, GC-MS analyses were performed by the equipment used in Section 3.5. All FAMEs could be successfully isolated on DB-WAX capillary columns (30 m × 0.25 mm × 0.25 µm; Agilent Technologies, Santa Clara, CA, USA). Helium served as the carrier gas at a constant flow velocity of 1.7 mL/min. One microliter isolate was injected into GC, and spitless mode was employed. The injector port temperature was kept at a constant temperature of 250 °C. The starting temperature of the column box was set at 60 °C for 5 min, then raised to 180 °C at a speed of 20 °C/min with a 6-min holding, then ramped to 200 °C at a speed of 2 °C/min with a 20-min holding, lastly raised to 230 °C at a speed of 4 °C/min with a 15.5-min holding. The FEMAs were quantitatively determined, and the corresponding fatty acid content was calculated on the basis of the corresponding conversion factor.

### 3.12. Data Analysis

The content of an odorant was received and listed as means ± standard deviation by utilizing Microsoft Excel 2021.

## 4. Conclusions

The thorough characterization of the potent odorants in SDEY and RDEY is provided in the present study. Twenty-four odorants with FD factors ranging from 3 to 6561 were screened by AEDA, yielding 17 odorants with OAVs > 1 that were classified as powerful odorants out of 45 volatiles. The numbers and contents of odor-active compositions and potent odorants in RDEY were more than those in SDEY. These newly formed odorants were associated with MR; there were very few odorants derived from fat oxidation. Acetoin was the most PO in SDEY; the more POs in RDEY included 2-ethyl-3,6-dimethylpyrazine, acetoin, 2-acetyl-3-methylthiophene, dihydro-4-hydroxy-2(3H)-furanone, etc. In the following study, the odor-reconstitution tests and omission experiments will be conducted to prove the results above and better understand the contribution of every potent odorant to the overall odor profiles of SDEY and RDEY.

## Figures and Tables

**Table 1:** Volatile compounds identified in salted duck egg yolk (SDEY) and roasted duck egg yolk (RDEY).

No.	Compound	CAS	RI		Samples		Identification ^a^
			DB-Wax	HP-5	SDEY	RDEY	
Pyrazines (7)							
**1**	Pyrazine	290-37-9	1199	723	− ^b^	+ ^c^	MS, RI, S, O
**2**	Methylpyrazine	109-08-0	/ ^d^	816	−	+	MS, RI, S
**3**	2,5-Dimethylpyrazine	123-32-0	1309	905	−	+	MS, RI, S, O
**4**	2,6-Dimethylpyrazine	108-50-9	1316	/	−	+	MS, RI, S, O
**5**	Ethylpyrazine	13925-00-3	1321	/	−	+	MS, RI, S
**6**	2-Ethyl-5-methylpyrazine	13360-64-0	1379	996	−	+	MS, RI, S, O
**7**	2-Ethyl-3,6-dimethylpyrazine	13360-65-1	1433	1073	−	+	MS, RI, S, O
Containing thiophene ring compounds (7)							
**1**	Thiophene	110-02-1	1014	/	−	+	MS, RI, S
**2**	2-Methylthiophene	554-14-3	1081	762	−	+	MS, RI, S, O
**3**	Dihydro-3-(2*H*)-thiophenone	1003-04-9	/	945	−	+	MS, RI
**4**	Dihydro-2-methyl-3(2*H*)-thiophenone	13679-85-1	1509	983	−	+	MS, RI, S, O
**5**	3-Acetylthiophene	1468-83-3	1759	/	−	+	MS, S
**6**	2-Acetyl-3-methylthiophene	13679-72-6	1851	1150	−	+	MS, S, O
**7**	2-Thiophenemethanol	636-72-6	1930	/	−	+	MS, RI, S
Containing furan ring compounds (7)							
**1**	2-Pentylfuran	3777-69-3	1222	986	−	+	MS, RI, S
**2**	2-Furanmethanol	98-00-0	1652	859	−	+	MS, RI, S, O
**3**	5-methyl-2-furanmethanol	3857-25-8	1710	/	−	+	MS, RI, S, O
**4**	3-Hydroxy-4,4-dimethyldihydro-2(3*H*)-furanone	79-50-5	2015	1032	−	+	MS, S, O
**5**	Dihydro-5-hydroxymethyl-2(3*H*)-furanone	32780-06-6	2479	1202	+	+	MS, S, O
**6**	Dihydro-4-hydroxy-2-(3*H*)-furanone	5469-16-9	2595	1173	+	+	MS, RI, S, O
**7**	7,9-Di-tert-butyl-1-oxaspiro(4,5)deca-6,9-diene-2,8-dione	82304-66-3	2699	1920	+	+	MS, RI, S
Alcohols (5)							
**1**	*β*-Butoxyethanol	111-76-2	1392	/	+	+	MS, RI, S, O
**2**	1-octen-3-ol	3391-86-4	1442	/	+	−	MS, RI, S, O
**3**	2-Ethyl-1-hexanol	104-76-7	1482	/	+	+	MS, RI, S, O
**4**	Carbitol	111-90-0	1610	/	+	+	MS, RI, S, O
**5**	Phenylethyl alcohol	60-12-8	1898	/	+	+	MS, RI, S
Aldehydes (4)							
**1**	Hexanal	66-25-1	1073	790	+	+	MS, RI, S
**2**	Nonanal	124-19-6	1382	1097	+	+	MS, RI, S
**3**	Benzaldehyde	100-52-7	1503	/	−	+	MS, RI, S
**4**	(*Z*)-9-Octadecenal	2423-10-1	2364	/	−	+	MS, S, O
Ketones (4)							
**1**	Acetoin	513-86-0	1270	/	+	+	MS, RI, S, O
**2**	1-Hydroxy-2-propanone	116-09-6	1286	/	−	+	MS, RI, S, O
**3**	2-Cyclopenten-1-one	930-30-3	1338	828	+	+	MS, RI, S
**4**	2-Hydroxy-3-methyl-2-cyclopenten-1-one	80-71-7	1814	/	−	+	MS, RI, S
Acids (3)							
**1**	2-Hydroxy-2-methylpropanoic acid	594-61-6	1327	/	−	+	MS, S
**2**	Acetic acid	64-19-7	1435	/	+	+	MS, RI, S, O
**3**	n-Hexadecanoic acid	57-10-3	/	1950	+	+	MS, RI, S
Phenols (2)							
**1**	Phenol	108-95-2	/	1990	+	+	MS, RI, S
**2**	2,4-Di-tert-butylphenol	96-76-4	2300	1504	+	+	MS, RI, S, O
Ester (1)							
**1**	Dibutyl phthalate	84-74-2	2694	/	−	+	MS, RI, S
Other heterocycle compounds (3)							
**1**	Pyrrole	109-97-7	1499	/	−	+	MS, RI, S
**2**	2-Acetylthiazole	24295-03-2	1633	1014	−	+	MS, RI, S, O
**3**	Indole	120-72-9	2435	/	+	+	MS, RI, S, O
Other sulfur-containing compounds (2)							
**1**	meta-Methoxybenzenethiol	15570-12-4	1720	/	−	+	MS, S
**2**	Dimethyl sulfone	67-71-0	1881	/	+	−	MS, S
Total: 45							

^a^ Identification: MS means identification by comparing with the NIST 14 mass spectra database. RI means identification by retention index. O means identification by odor characteristic. S means confirmed by authentic standards. ^b^ − means the odorant is not identified. ^c^ + means the odorant is identified. ^d^ / means the odorant is not isolated on the column.

**Table 2:** Odor-active compounds screened and their flavor-dilution factors.

No.	Compounds	Odor Characteristics ^a^	FD Factor ^b^	
			SDEY	RDEY
**1**	2-methylthiophene	meaty, roasty	-	9
**2**	pyrazine	nut	-	9
**3**	acetoin	butter, creamy	81	27
**4**	1-hydroxy-2-propanone	butter, malty	-	6561
**5**	2,5-dimethylpyrazine	cocoa, meaty, nutty	-	9
**6**	2,6-dimethylpyrazine	cocoa, meaty, roasted	-	9
**7**	2-ethyl-5-methylpyrazine	fruit, green, nutty	-	9
**8**	*β*-butoxyethanol	sweet	81	3
**9**	2-ethyl-3,6-dimethylpyrazine	potato, roasted	-	243
**10**	acetic acid	acid, sour	9	3
**11**	1-octen-3-ol	mushroom, earthy	729	-
**12**	2-ethyl-1-hexanol	green, rose, fruity	81	243
**13**	dihydro-2-methyl-3(2*H*)-thiophenone	cabbage, must, onion	-	3
**14**	Carbitol	sweet, burnt	27	243
**15**	2-acetylthiazole	nut, roasted, sulfur	-	3
**16**	2-furanmethanol	burnt, caramel, cooked	-	243
**17**	5-methyl-2-furanmethanol	roasted, sweet, caramel	-	81
**18**	2-acetyl-3-methylthiophene	phenolic	-	243
**19**	3-hydroxy-4,4-dimethyldihydro-2(3*H*)-furanone	sweet, caramel	-	6561
**20**	2,4-di-tert-butylphenol	phenolic, leather	243	729
**21**	(*Z*)-9-octadecenal	fatty	-	729
**22**	indole	fecal, jasmine	9	243
**23**	dihydro-5-hydroxymethyl-2(3*H*)-furanone	burnt	729	2187
**24**	dihydro-4-hydroxy-2-(3*H*)-furanone	caramel	9	243

^a^ Odor characteristic is sniffed by GC-O. ^b^ FD factor means flavor-dilution factor measured on a DB-Wax column.

**Table 3:** Free amino acid composition and concentration.

No.	Name	Concentrations (μg/g)	
		SDEY	RDEY
**1**	Asp	605.19 ± 4.53	506.77 ± 39.04
**2**	Glu	1332.06 ± 16.99	1353.94 ± 73.93
**3**	Ser	591.83 ± 13.15	872.95 ± 54.73
**4**	Gly	238.9 ± 7.51	442.02 ± 30.82
**5**	Thr	453.43 ± 8.5	605.81 ± 22.84
**6**	Arg	805.09 ± 63.84	736.47 ± 42.14
**7**	Ala	588.78 ± 30.24	646.28 ± 26.18
**8**	Tyr	860.7 ± 136.42	1054.23 ± 92.34
**9**	Val	521.67 ± 10.43	3063.92 ± 51.74
**10**	Met	381.99 ± 4.62	675.51 ± 12.15
**11**	Phe	423.32 ± 19.7	910.22 ± 47.9
**12**	Lys	390.77 ± 9.06	482.01 ± 10.81
**13**	Leu	1069 ± 28.23	1262.79 ± 31.4
**14**	Pro	96.35 ± 14.83	816.3 ± 45.9
Total		8359.08	13,429.23

**Table 4:** The concentrations of fatty acids in duck egg yolk oil.

No.	Fatty Acids	Concentrations (mg/kg)	
		SDEY Oil	RDEY Oil
**1**	Lauric acid (C12:0)	195.81 ± 1.44	196.28 ± 5.98
**2**	Myristic acid (C14:0)	4675.05 ± 0.08	4302.53 ± 101.1
**3**	Myristoleic acid (C14:1)	100.73 ± 1.27	87.94 ± 1.88
**4**	*n*-Pentadecanoic acid (C15:0)	381.3 ± 0.51	363.68 ± 5.09
**5**	Palmitic acid (C16:0)	240,713.5 ± 272.11	233,379.95 ± 11,532.77
**6**	Palmitoleic acid (C16:1)	19,566.61 ± 65.31	12,834.28 ± 1117.97
**7**	Margaric acid (C17:0)	14.1 ± 0.01	12.15 ± 1.14
**8**	*cis*-10-Heptadecenoic acid (C17:1)	10.43 ± 0.17	8.5 ± 0.39
**9**	Oleic acid (C18:1n9c)	248,608.41 ± 30,523.47	246,942.24 ± 37,745.7
**10**	Linoleic acid (C18:2n6c)	40,767.13 ± 108.92	74,238.73 ± 2636.37
**11**	*γ*-Linolenic acid (C18:3n6)	1486.76 ± 163.51	1755.16 ± 17.3
**12**	Linolenic acid (C18:3n3)	1453.39 ± 103.08	2598.92 ± 205.41
**13**	Arachidic acid (C20:0)	521.19 ± 27.78	2.51 ± 1.92
**14**	11,14-Eicosadienoic acid (C20:2)	1191.17 ± 9.93	235.36 ± 31.11
**15**	Arachidonic acid (C20:4n6)	5509 ± 1.46	4142.26 ± 285.4
**16**	11,14,17-Eicosatrienoic acid (C20:3n3)	21.48 ± 0.08	32.72 ± 3.83
**17**	(all-*Z*)-4,7,10,13,16,19-Docosahexaenoic acid (C22:6n3)	78.24 ± 3.52	70.78 ± 27.29
	Total	565,294.30	581,203.99

**Table 5:** Concentrations of important odor-active compounds.

No.	Compounds	IS ^a^	*f* ^b^	Concentrations (mg/kg)	
				SDEY	RDEY
**1**	2-methylthiophene	IS1	0.43	-	5.9 ± 0.26
**2**	pyrazine	IS1	0.67	-	441.54 ± 2.05
**3**	acetoin	IS1	1.34	136.31 ± 0.3	70.77 ± 3.71
**4**	1-hydroxy-2-propanone	IS1	1.31	-	1072.91 ± 70.62
**5**	2,5-dimethylpyrazine	IS1	0.98	-	45.73 ± 1.95
**6**	2,6-dimethylpyrazine	IS2	0.84	-	14.45 ± 0.26
**7**	2-ethyl-5-methylpyrazine	IS2	2.46	-	7.82 ± 0.75
**8**	*β*-butoxyethanol	IS2	0.50	7.73 ± 0.29	-
**9**	2-ethyl-3,6-dimethylpyrazine	IS2	3.46	-	126.99 ± 1.44
**10**	acetic acid	IS2	1.00	28.2 ± 0.36	-
**11**	1-octen-3-ol	IS2	0.46	3.58 ± 0.01	-
**12**	2-ethyl-1-hexanol	IS2	1.27	8.22 ± 0.29	18.59 ± 0.21
**14**	carbitol	IS2	3.41	2.96 ± 0.18	20.81 ± 0.62
**16**	2-furanmethanol	IS2	4.22	-	60.28 ± 1.12
**17**	5-methyl-2-furanmethanol	IS2	2.70	-	26.33 ± 0.54
**18**	2-acetyl-3-methylthiophene	IS3	31.05	-	262.98 ± 47.91
**19**	3-hydroxy-4,4-dimethyldihydro-2(3*H*)-furanone	IS3	1.00	-	41.61 ± 0.94
**20**	2,4-di-tert-butylphenol	IS3	0.62	66.7 ± 0.34	216.86 ± 4.56
**21**	(*Z*)-9-octadecenal	IS3	1.00	-	2.03 ± 0.19
**22**	indole	IS3	1.07	4.37 ± 0.27	26.38 ± 9.46
**23**	dihydro-5-hydroxymethyl-2(3*H*)-furanone	IS3	0.51	123.77 ± 1.29	628.64 ± 10.88
**24**	dihydro-4-hydroxy-2(3*H*)-furanone	IS3	5.75	23.60 ± 1.26	2613.04 ± 54.75

^a^ IS means internal standard; IS1, IS2, and IS3 stand for 2-methyl-3-heptanone, 2-octanol, and 2-isopropylphenol, respectively. ^b^ *f* means the relative correction factor.

**Table 6:** Odor activity value of important odor-active compounds.

No.	Compounds	Threshold mg/kg ^a^	OAV ^b^	
			SDEY	RDEY
**3**	acetoin	0.014	9737	5055
**11**	1-octen-3-ol	0.015	238	-
**20**	2,4-di-tert-butylphenol	0.5	113	434
**24**	dihydro-4-hydroxy-2(3*H*)-furanone	3.741	33	698
**22**	indole	0.3	14	88
**8**	*β*-butoxyethanol	0.88	8	-
**12**	2-ethyl-1-hexanol	1.5	< 1	12
**10**	acetic acid	99	< 1	-
**14**	carbitol	1.6	< 1	13
**9**	2-ethyl-3,6-dimethylpyrazine	0.0086	-	14,766
**18**	2-acetyl-3-methylthiophene	0.1	-	2630
**2**	pyrazine	2.5	-	177
**4**	1-hydroxy-2-propanone	10	-	107
**7**	2-ethyl-5-methylpyrazine	0.1	-	78
**5**	2,5-dimethylpyrazine	1.75	-	26
**6**	2,6-dimethylpyrazine	0.718	-	20
**16**	2-furanmethanol	4.5	-	14
**1**	2-methylthiophene	3	-	2
**17**	5-methyl-2-furanmethanol	/	-	/
**19**	3-hydroxy-4,4-dimethyldihydro-2(3*H*)-furanone	/	-	/
**21**	(*Z*)-9-octadecenal	/	-	/
**23**	dihydro-5-hydroxymethyl-2(3*H*)-furanone	/	/	/

^a^ odor-detection thresholds in water from Van Gemert (2011) [28]. ^b^ OAV means odor activity value (ratio of concentration to odor threshold).

## Data Availability

The data are available upon reasonable request.
